# Cold-shock eliminates female nucleus in fertilized eggs to induce androgenesis in the loach (*Misgurnus anguillicaudatus*), a teleost fish

**DOI:** 10.1186/1472-6750-11-116

**Published:** 2011-11-29

**Authors:** Kagayaki Morishima, Takafumi Fujimoto, Mami Sato, Ayako Kawae, Yan Zhao, Etsuro Yamaha, Katsutoshi Arai

**Affiliations:** 1 Laboratory of Aquaculture Genetics and Genomics, Faculty and Graduate School of Fisheries Sciences, Hokkaido University, 3-1-1, Minato, Hakodate, Hokkaido, 041-8611, Japan; 2 Nanae Fresh-Water Laboratory, Field Science Center for Northern Biosphere, Hokkaido University, 2-9-1 Sakura-machi, Nanae, Hokkaido, 041-1105, Japan

## Abstract

**Background:**

Androgenesis (all-male inheritance) is generally induced by means of irradiating the eggs to inactivate the maternal genome, followed by fertilization with normal sperm. In fish, the conventional technique for induced androgenesis has been applied for rapid fixation to traits, recovery of cryopreserved genotypes, sex-control, etc. A new method of androgenesis that eliminates the need to irradiate the egg was proposed using the loach, *Misgurnus anguillicaudatus *(a teleost fish).

**Results:**

When the eggs of wild-type females were fertilized with sperm of albino or orange phenotype males and cold-shocked at 0 to 3°C for 60 min duration just after fertilization, generally more than 30% (with a peak of 100%) of the hatched progeny were androgenotes. While a few of them were the normal diploid, most of them turned out to be abnormal haploid. All-male inheritance was verified by the expression of the recessive color trait (albino or orange) and microsatellite genotypes comprising only paternally derived alleles. Nuclear behavior after the cold-shock treatment was traced by microscopic observation of DAPI (4'6-diamidino-2-phenylindole)-stained samples and hematoxylin-eosin stained histological sections, and the extrusion of egg (maternal) nucleus was observed in eggs treated in the optimum timing.

**Conclusion:**

In this paper, we demonstrate that cold-shock treatment (at 0 and 3°C) of loach eggs for 60 min just after fertilization successfully induces androgenetic haploid development. The most likely mechanism of cold-shock induced androgenesis is an elimination of the egg nucleus together along with the second polar body and subsequent development of a decondensed sperm nucleus or male pronucleus.

## Background

Androgenesis may be defined as uniparental reproduction without any genetic contribution from the maternally derived nucleus. In fish, artificial androgenesis has been induced by fertilization of genetically inactivated eggs with normal spermatozoa [1-4]. Genetic inactivation of egg nucleus has typically been achieved by means of irradiating the eggs with gamma- and X-rays, but more recently it has been shown that the egg nucleus can be successfully inactivated using ultraviolet (UV) irradiation, especially in fish with relatively smaller egg sizes [1-4]. In aquaculture, cloning, rapid fixation of genotypes and sex-control (especially using super-male with YY genotype) are usually proposed by using genomes of completely homozygous doubled haploids, which are induced by chromosome duplication (endomitosis) at the first cleavage after the initiation of androgenetic development of haploid embryos [2,3]. Duplication of chromosomes in haploids is induced by temperature or pressure shock applied at the optimum timing of the pro-metaphase of the first mitotic cell division [5]. Homozygous clones have been produced in several fish species such as common carp [6], Nile tilapia [7], amago salmon [8], and rainbow trout [9]. Androgenesis is also regarded as a useful approach to recover genotypes from cryopreserved sperm of unique or endangered species [10]. Nagoya et al. [11] successfully produced viable androgenetic diploid amago salmon with gamma-irradiated eggs and subsequent dispermy fertilization using fusion of spermatozoa by PEG (polyethylene glycol). Similar attempts using induced androgenesis and fused spermatozoa or dispermy have also been reported in rainbow trout [12], barb [13], tetra [14] and sturgeon [15].

In clonally reproducing teleosts such as crucian carp and loach, a sperm nucleus that intrudes into an unreduced egg is never decondensed to form the male pronucleus, and gynogenetic development proceeds without any genetic contribution from the paternal genome [16,17]. Spontaneous gynogenesis (all-female inheritance) is fairly common in lower vertebrates, including teleosts [18,19]. In contrast, spontaneous androgenetic individuals (all-male inheritance) have never been reported in vertebrates, although the phenomenon is seen in a few invertebrates including the hermaphrodite triploid clam [20,21], stick insect [22] and laboratory *Drosophila *hybrid [23]. On the other hand, in a large number of artificial triploidization studies in fish, an occurrence of abnormal embryos with haploid-like external appearance has been often reported in the treatment shortly after fertilization. In experiments to produce triploid salmonids, haploid embryos were cytogenetically recognized in heat-shocked groups [24,25] and pressure-shocked groups [25]. Such an incidence of haploid-like progeny was also reported in experiments to induce triploidy by inhibiting the second polar body release with cold-shock treatments shortly after fertilization in stickleback [26,27], common carp [28] and loach [29]. Although the origin of such unusual haploid-like progeny has not been well examined in these fish species, Ueda and Aoki [30] cytogenetically confirmed one androgenetic haploid embryo out of ten diploid hybrid embryos developing from cold shock (0°C for 60 min) at 2 min after fertilization of eggs from *Rhodeus ocellatus ocellatus *(Rosy bitterling) with sperm from *Acheilognathus rhombea *(Kanehira bitterling). They suggested induction as a possible mechanism for androgenesis by temperature control of fertilized eggs. However, further studies are required since their results are from a single trial and a small sample size.

Since it is difficult to achieve perfect elimination of chromosome fragments of egg nucleus in androgenetic induction by the regular method of egg irradiation with UV [4], temperature shock of eggs shortly after fertilization may provide a new, easy and simple method to induce androgenesis in fish species and presumably in other vertebrate species. However, at present, treatment conditions to induce androgenesis have not been optimized in any fish species and mechanisms underlying such a temperature-induced androgenesis have not been examined yet.

In the present study, we optimized the cold-shock condition among different temperature (0, 3, 6 and 9°C) for 60 min duration just after fertilization to induce androgenetic progeny in the loach using albino color phenotype (recessive trait) as the paternal genetic marker. Abnormal morphological characteristic (referred to as the haploid syndrome) is another marker of haploid development. Ploidy (haploidy, diploidy, triploidy or others) was determined by DNA content flow cytometry, and microsatellite genotypes were analyzed to confirm all-paternal inheritance in the putative androgenetic progeny. Next, in the selected cold-shock condition at 3°C for 60 min duration shortly after fertilization, six crosses were conducted using orange phenotype (recessive trait) as the paternal color marker. In cold-shocked eggs and resultant embryos, cytological and histological observations were then performed to disclose the mechanism responsible for the initiation of androgenetic development after the cold-shock treatment.

## Results

### Cold-shock treatments and phenotypes of the resultant progeny

**Figure 1:**
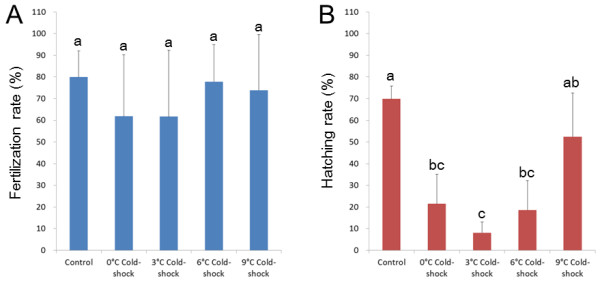
**Fertilization (A) and hatching rates (B) relative to total eggs used in control and 0, 3, 6 and 9°C cold-shocked groups from five different crosses**. Histograms and bars denote means and SD, respectively. Different letters mean significant differences (*P *< 0.05).

**Figure 2:**
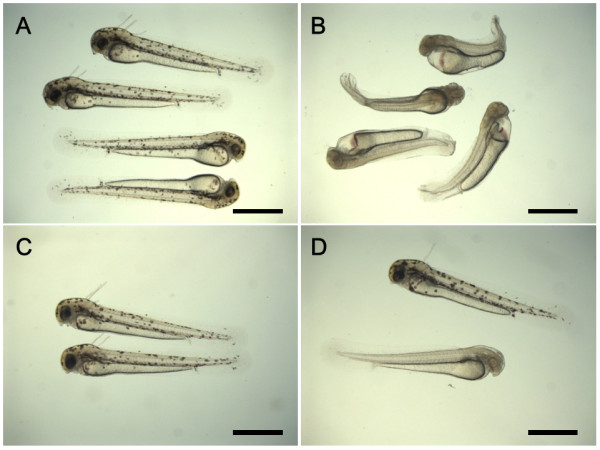
**External appearance of larvae developing from the control and cold-shocked eggs in the loach**. A: Wild-type fry in the control group with normal external appearance; B: Albino fry from the 0°C cold-shocked group with abnormal external appearance; C: Wild-type fry from the 0°C cold-shocked group with normal external appearance; D: Wild-type (upper) and albino (lower) fry from the 3°C cold-shocked group with normal external appearance. Scales denote 1 mm.

Eggs were cold-shocked at 0, 3, 6 or 9°C just after fertilization to optimize temperature conditions in the first experiments comprising five crosses (A to E). The percentages of fertilized eggs were generally high and were not significantly different among groups including control (Figure 1A). Groups cold-shocked at 0, 3, and 6°C gave significantly lower hatching rates (8.1% - 21.5%) when compared with control (69.9%) (Figure 1B). Hatching rate (52.4%) of 9°C cold-shock group was not significantly different from the control (Figure 1B). Normal wild-type with melanophores appeared in control (Figure 2A), while cold-shock treatments resulted in the significant occurrence of the abnormal albino phenotype (a color marker for all-paternal inheritance, or androgenesis) (Figure 2B), besides the dominant normal wild-type (Figure 2C). Very few normal albino fry appeared in cold-shocked groups (Figure 2D).

**Figure 3:**
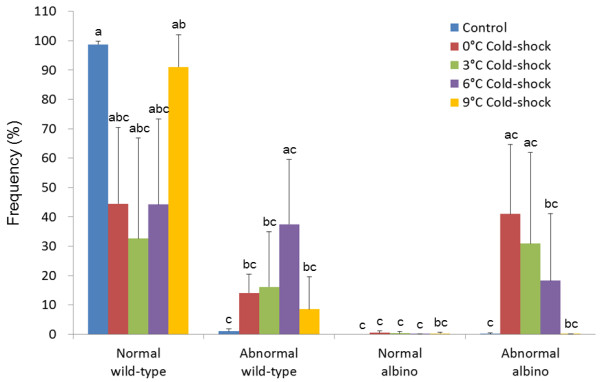
**Frequencies of four kinds of phenotypes, normal wild-type, abnormal wild-type, normal albino and abnormal albino in hatched fry from control and 0, 3, 6 and 9°C cold-shocked groups from five different crosses**. Histograms and bars denote means and SD, respectively. Different letters mean significant differences (*P *< 0.05).

As shown in Figure 3, most hatched larvae were normal wild-type in control (98.8%) and 9°C cold-shocked group (91.1%). Frequencies of normal wild-type decreased in 0, 3 and 6°C cold-shocked groups. Instead, abnormal wild-type larvae occurred in cold-shocked groups. High frequencies of abnormal albino fry were recorded in groups cold-shocked at 0°C (41.0%) and 3°C (33.9%). Cold-shock at 6°C occasionally resulted in relatively lower rates of the abnormal albino (18.8%). On the other hand, cold-shock at 9°C resulted in a very low percentage of albinos (0.1%), if any. It must also be mentioned that in addition, a very small number of normal albino fry and abnormal albinos were seen in the cold-shocked groups (0.1 -0.3%) and control groups (0.2%), respectively.

### Ploidy determination in progeny from cold-shock treatments

**Figure 4:**
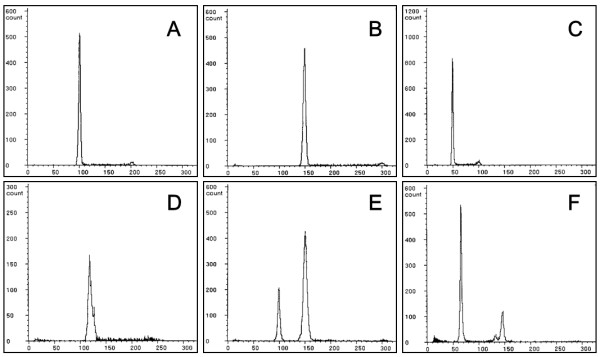
**Relative DNA contents of fry developing from the control and cold-shocked groups**. A: Wild-type diploid of the control group; B: Wild-type triploid from the 0°C cold-shocked group; C: Albino haploid from the 0°C cold-shocked group; D: Hyper diploid showing wild-type phenotype from the 0°C cold-shocked group; E: Euploid mosaic (diploid and triploid) with wild-type phenotype from the 6°C cold-shocked group; F: Aneuploid mosaic (1.3n-2.9n) with wild-type phenotype from the 9°C cold-shocked group.

Ploidy of normal wild-type, abnormal wild-type, normal albino and abnormal albino progeny from control and cold-shock treatments in five crosses (A to E) was assessed by flow cytometry (Table 1 Figure 4). In the controls, all the normal wild-type progeny were diploid (Figure 4A), whereas a few abnormal wild-type progeny contained haploid, diploid and hypodiploid progeny. A small number of abnormal albino phenotypes also appeared, but these were aneuploids with hypodiploid DNA content. In normal wild-type progeny from 0, 3 and 6°C cold-shock treatments, both diploid and triploid (Figure 4B) progeny appeared, while those from 9°C group included only diploid individuals. Among the abnormal wild-type progeny from cold-shock treatments, haploid, diploid, triploid, aneuploid (Figure 4D) and mosaic (Figure 4EF) progeny appeared. All the abnormal albino progeny that appeared in 0, 3 and 6°C cold-shock treatments were haploid (Figure 4C), while very few normal albino progeny were diploid. Consequently, cold-shock just after fertilization often resulted in androgenetic haploid development, as shown by the appearance of abnormal albino phenotypes and haploid status in the progeny. In general, a high percentage of albino phenotype (putative androgenotes) was detected from the cold-shock treatment at 0 to 3°C.

**Table 1:** Ploidy of fry from the control and cold-shocked eggs from the first experiment comprising five crosses (A to E).

Treatment	Phenotype	Morphology	No. of fry	Ploidy status							
				
				1n	2n	3n	6n	Hypo-2n	Hyper-2n	Euploid mosaic	Aneuploid mosaic
Control	Wild-type	Normal	74	0	74	0	0	0	0	0	0
	Wild-type	Abnormal	7	1	1	0	0	5^1^	0	0	0
	Albino	Abnormal	2	0	0	0	0	2^2^	0	0	0
0°C Cold-shock	Wild-type	Normal	71	0	18	53	0	0	0	0	0
	Wild type	Abnormal	38	5	18	5	0	5^3^	2^4^	2^5^	1^6^
	Albino	Normal	1	0	1	0	0	0	0	0	0
	Albino	Abnormal	83	83	0	0	0	0	0	0	0
3°C Cold-shock	Wild-type	Normal	32	0	2	29	1	0	0	0	0
	Wild-type	Abnormal	24	2	6	5	0	0	10^7^	0	1^8^
	Albino	Abnormal	62	62	0	0	0	0	0	0	0
6°C Cold-shock	Wild-type	Normal	46	0	26	14	0	0	0	4^9^	2^10^
	Wild-type	Abnormal	35	10	7	2	0	4^11^	6^12^	1^13^	5^14^
	Albino	Normal	1	0	1	0	0	0	0	0	0
	Albino	Abnormal	51	51	0	0	0	0	0	0	0
9°C Cold-shock	Wild-type	Normal	40	0	40	0	0	0	0	0	0
	Wild-type	Abnormal	4	0	2	0	0	0	1^15^	0	1^16^

^1 ^1.8n(2 individuals), 1.7n(1), 1.6n(1), 1.4n(1); ^2 ^1.8n(1), 1.6n(1); ^3 ^1.8n(3), 1.9n (1), 1.6n(1); ^4 ^2.1n(1), 2.5n(1); ^5 ^1n-3n(1), 1n-5n (1); ^6 ^2n-2.2n (1); ^7 ^2.2n(2), 2.3n(2), 2.4n(1), 2.6n(2), 2.8n(1), 2.9n(2); ^8 ^2.8n-3.2n; ^9 ^1n-2n(2), 2n-3n(2); ^10 ^2n-2.1n (1), 1.9n-2.9n(1); ^11 ^1.9n(1), 1.8n(1), 1.7n(1), 1.3n(1); ^12 ^2.8n(1), 2.7n(1), 2.6n(1), 2.5n(1), 2.4n(1), 2.2n(1); ^13 ^2n-3n(1); ^14 ^1.8n-2n(1), 2.6n-3n(1), 1.7n-2n(1), 2n-2.3n(1), 1n-1.1n (1); ^15 ^2.2n(1);^16 ^1.3n-2.9n (1).

### Genetic verification of androgenesis by microsatellite genotyping

Microsatellite genotyping was carried out at *Mac60 *and *63 *loci [31] from normal wild-type diploid progeny from the control (*n *= 9), abnormal albino haploid progeny (*n *= 7), normal albino diploid progeny (*n *= 1), normal wild-type triploid progeny (*n *= 9), and abnormal wild type haploid progeny (*n *= 6) developing from the cold-shocked eggs in the cross A (Table 2). Although maternally and paternally derived alleles were segregated in normal wild-type diploid progeny according to Mendelian laws of inheritance, only paternal alleles were detected in abnormal haploid and normal diploid progeny with albino phenotype. Therefore, albino progeny were concluded to be androgenotes. Triploid wild-type progeny included two alleles of the mother and one allele of the father. Abnormal haploid progeny with wild-type phenotype included only maternally derived alleles and thus they are concluded to be sporadic gynogenetic haploid progeny.

**Table 2:** Microsatellite *Mac 60 *and *63 *genotypes of normal and abnormal progeny with wild or albino phenotypes developing from the control and cold-shocked eggs from a selected cross A in the first experiment.

Locus (LG)^1^	Female	Male	Control				Cold Shock							
			
			Wild-type, Normal				Albino, Abnormal		Albino, Normal		Wild-type, Normal		Wild-type, Abnormal	
			
			Diploid				Haploid		Diploid		Triploid		Haploid	
			
	(*a/b*)	(*c/d*)	*a/c*	*a/d*	*b/c*	*b/d*	*c*	*d*	*cc*	*dd*	*abc*	*abd*	*a*	*B*
*Mac60 *(LG4)	*128/135*	*142/148*	4	2	*2*	1	3	4	1	0	5	4	3	3
*Mac63 *(LG4)	*125/138*	*165/187*	3	2	2	2	5	2	0	1	5	4	3	3

^1 ^Linkage group [31]

### Production of androgenetic progeny by 3°C cold-shock

**Figure 5:**
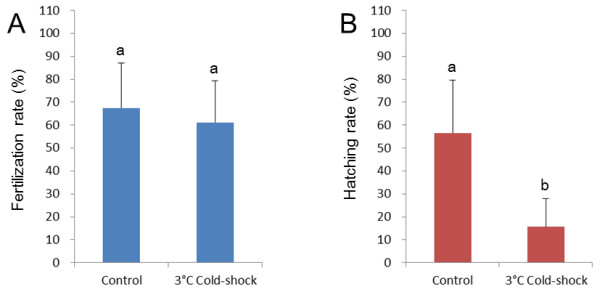
**Fertilization (A) and hatching rates (B) relative to total eggs used in control and 3°C cold-shocked groups from six different crosses**. Histograms and bars denote means and SD, respectively. Different letters mean significant differences (*P *< 0.05).

**Figure 6:**
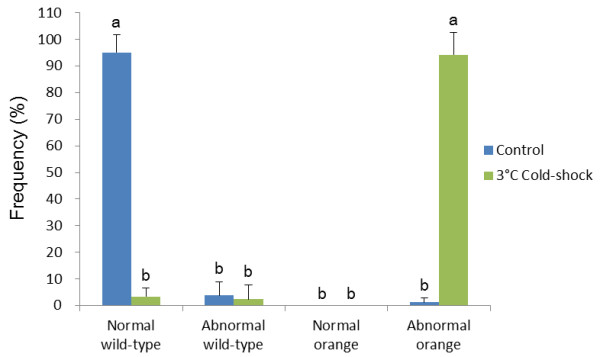
**Frequencies of four kinds of phenotypes, normal wild-type, abnormal wild-type, normal albino and abnormal albino in hatched fry from control and 3°C cold-shocked groups from six different crosses**. Histograms and bars denote means and SD, respectively. Different letters mean significant differences (*P *< 0.05).

Using a specific condition of the cold-shock (3°C for 60 min duration just after fertilization), eggs of wild-type females inseminated with sperm of orange (recessive trait) males were followed in the second experiment comprising six crosses (F to K). Fertilization rates were not significantly different between control (67.5%) and cold-shocked group (61.1%) (Figure 5A). Significantly lower hatching rate (15.7%) was recorded from cold-shocked group when compared to the control (56.5%) (Figure 5B). High percentage of abnormal progeny with orange phenotype (94.3%) was recorded only in cold-shocked groups. In the control, most progeny (95.1%) were normal exhibiting the wild-type phenotype, with a very few abnormal orange (1.2%) and abnormal wild-type progeny (3.4%) (Figure 6).

Flow cytometry studies (Table 3) showed that a majority of normal wild-type progeny (133/134) and abnormal wild type progeny (12/18) in the control were diploid and others were mosaics. On the other hand, both diploid (4/11) and triploid (7/11) progeny appeared in normal wild-type progeny from cold-shock treatments. Abnormal wild-type progeny in cold-shock were diploid (1/3) and aneuploid (2/3). A large number of abnormal orange progeny in cold-shocked groups (108/109) and a small number of abnormal orange progeny in the control groups (3/5) were haploid except for a few aneuploids.

**Table 3:** Ploidy of fry from the control and cold-shocked eggs in the second experiment including six crosses (F to K).

Treatment	Phenotype	Morphology	Fry				Ploidy status			
				
			**no**.	1n	2n	3n	Hypo-2n	Hyper-2n	Euploid Mosaic	Aneuploid Mosaic
Control	Wild-type	Normal	134	0	133	0	0	0	1^1^	0
	Wild-type	Abnormal	18	0	12	0	0	0	5^2^	1^3^
	Orange	Abnormal	5	3	0	0	2^4^	0	0	0
3°C Cold shock	Wild-type	Normal	11	0	4	7	0	0	0	0
	Wild-type	Abnormal	3	0	1	0	0	2^5^	0	0
	Orange	Abnormal	109	108	0	0	0	1^6^	0	0

^1 ^1n-2n (1 individual); ^2 ^1n-2n (3), 1n-3n (1), 1n-2n-3n(1); ^3 ^1.8n-2n (1); ^4 ^1.8n (2); ^5 ^2.6n (2); ^6 ^2.7n (1).

Normal diploid progeny with wild-type phenotype in the control and abnormal haploid progeny with orange color in the cold-shocked group from the two crosses (I and K) were genotyped at three independent microsatellite loci [31], *Mac 60, 402 *and *519 *(Table 4). Maternally and paternally derived alleles were segregated in the diploid progeny from the control, while only the paternally derived allele was detected in orange haploid progeny. These results indicate that abnormal orange progeny were androgenetic haploid developing from cold-shocked eggs.

**Table 4:** Microsatellite genotyping using *Mac 60, 402 *and *519 *loci of normal and abnormal progeny with wild or orange phenotypes developing from the control and cold-shocked eggs of two crosses (I and K) in the second experiment.

**Exp**.	Locus (LG)^1^	Female	Male	Control				Cold-shock	
				
				**Wild-type**, Normal				Orange, Abnormal	
				
				Diploid				Haploid	
				
		(*a/b *or *a/a*)	(*c/d *or *c/c*)	*a/c*	*a/d*	*b/c*	*b/d*	*c*	*d*
I	*Mac60*(LG4)	*126/132*	*124/124*	15	0	9	0	21	0
	*Mac402*(LG8)	*384/388*	*370/373*	4	4	12	3	13	8
	*Mac519*(LG11)	*309/326*	*254/344*	7	6	6	4	9	11
K	*Mac60*(LG4)	*131/133*	*124/124*	6	0	17	0	19	0
	*Mac402*(LG8)	*401/401*	*370/373*	13	9	0	0	14	5
	*Mac519*(LG11)	*277/309*	*254/344*	6	8	4	4	10	9

^1 ^Linkage group [31]

### Cytological stages of DAPI stained nucleus in cold-shocked embryos

**Figure 7:**
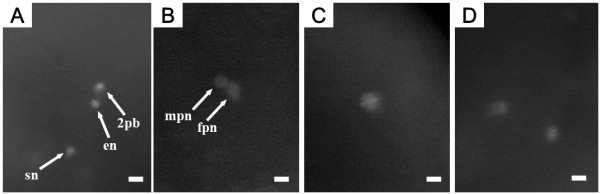
**DAPI-stained nuclear behavior in eggs after fertilization in the control**. A (10 min after fertilization; af): three condensed nuclei, sperm nucleus (sn), egg nucleus (en) and second polar body nucleus (2 pb); B (20 min af) two decondensed pronuclei, female pronucleus (fpn) and male pronucleus (mpn); C (30 min af) decondensed and fused one pronucleus; D (40 min af) anaphase of the first cleavage. Scale bars denotes 10 μm.

DAPI-stained nuclear behavior in the progeny from the control is shown in Figure 7 and Table 5. Two or three condensed nuclei were observed in these embryos 10 min after fertilization (af), presumably the sperm nucleus, egg nucleus and/or second polar body nucleus (Figure 7A, Table 5). At 20 min af, the putative egg and sperm nucleus decondensed to form the two pronuclei (female and male), respectively (Figure 7B, Table 5), after which, the two pronuclei fused to form a decondensed pronucleus or a zygotic nucleus at 30 min af (Figure 7C, Table 5). At 40 min af, first cleavage anaphase was observed (Figure 7D, Table 5), and the nucleus became difficult to detect at 50 min af. However, the cleavage furrow was detected and the embryos entered into the two-cell stage at 60 min af (Figure not shown, Table 5).

**Table 5:** Stages of DAPI-stained nuclear behavior in the control and cold-shocked eggs of the loach at specific time intervals after fertilization.

Stages	Time elapsed after fertilization (min)															
	
	Control						Cold-shock									
	
	10	20	30	40	50	60	10	30	60	70	80	90	100	110	120	130
1 condensed nucleus	0	0	0	0	0	0	5	3	6	11	6	6	1	4	1	0
2 condensed nuclei	4	0	0	0	0	0	12	16	15	5	11	4	3	1	1	0
3 condensed nuclei	4	0	0	0	0	0	3	0	0	8	3	0	1	0	0	0
2 condensed nuclei + 1 decondensed pronucleus	0	0	0	0	0	0	0	0	0	2	2	0	0	0	1	0
1 condensed nucleus + 1 decondensed pronucleus	0	0	0	0	0	0	0	0	0	0	5	5	1	0	0	0
2 decondensed pronuclei	0	6	1	0	0	0	0	0	0	0	2	1	0	0	0	0
1 decondensed pronucleus	0	3	4	1	1	0	0	0	0	0	2	3	1	0	1	0
First cleavage Anaphase	0	0	0	4	1	0	0	0	0	0	0	0	1	0	0	0
2-cell stage	0	0	0	0	1	11	0	0	0	0	0	0	0	0	3	9
Others ^1^	0	0	2	2	4	0	0	1	0	0	1	6	6	16	5	0
Total number of eggs observed	8	9	7	7	7	11	20	20	21	26	32	25	14	21	12	9

^1 ^cytological observation could not be made.

**Figure 8:**
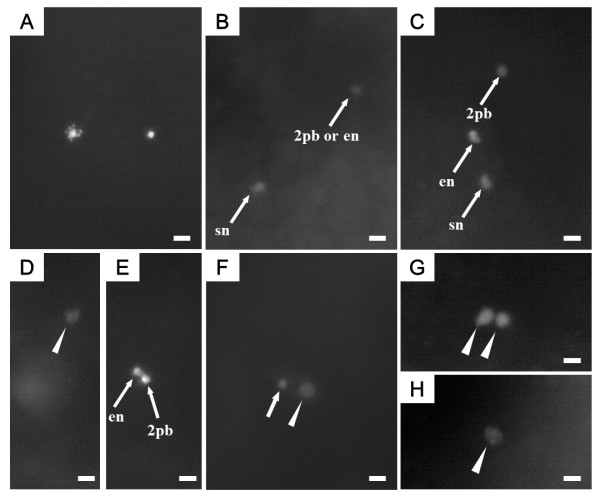
**DAPI-stained nuclear behavior in cold-shocked eggs after fertilization**. Arrows indicate condensed nuclei. Arrowheads indicate decondensed pronuclei. A (60 min after fertilization (af); just after the cold-shock treatment): two condensed nuclei, egg nucleus and sperm nucleus; B (70 min af): two condensed nuclei, sperm nucleus (sn) and egg nucleus (en) or second polar body nucleus (2 pb); C (80 min af): three condensed nuclei, sperm nucleus, egg nucleus and second polar body nucleus; D (70 min af): one decondensed male pronucleus, section from the same egg shown in E; E (70 min af): two condensed nuclei, egg nucleus and second polar body nucleus; F (90 min af): one condensed nucleus and one decondensed pronucleus; G (90 min af): two decondensed pronuclei; H (90 min af): one decondensed pronucleus. Scale bars denote 10 μm.

In the cold-shock treatment one to three condensed nuclei were observed within 60 min af, (Figure 8A, Table 5). When compared to control embryos, these nuclei were strongly condensed and heavily stained, and two (Figure 8B) and/or three (Figure 8C) condensed nuclei could still be seen until 120 min af. A decondensed pronucleus was firstly detected at 70 min af, together with two condensed nuclei (Figure 8D and 8E, Table 5). At 80 min af, embryos with one condensed nucleus and one decondensed pronucleus were seen (Figure 8F, Table 5), along with embryos with two decondensed pronuclei (Figure 4G) and those with only one decondensed pronuleus (Figure 8H, Table 5). Anaphase of the first cleavage was detected at 100 min af. At 100 and 110 min af, it was difficult to observe the behavior of the nucleus, but the two-cell stage was detected at 120 min af (Table 5).

### Histological observation of the nucleus in cold-shocked embryos

**Figure 9:**
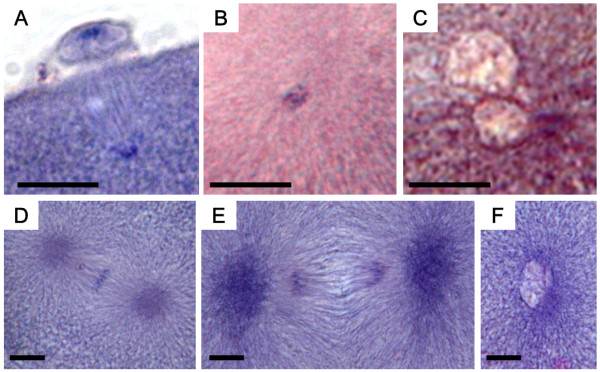
**Histological sections of control eggs after fertilization**. A (10 min after fertilization: af): second polar body released and egg nucleus present just underneath the polar body; B (10 min af): condensed sperm nucleus; C (20 min af): decondensed female and male pronuclei; D (30 min af): metaphase of first cleavage; E (40 min af): anaphase of first cleavage; F (50 min af): prophase of second cleavage. Scale bars denote 10 μm.

In control embryos, the second polar body was released at 10 min af (Table 6), and the polar body nucleus and female (egg) nucleus were both observed (Figure 9A). The female nucleus was located just underneath the second polar body nucleus in the egg, and the male (sperm) nucleus was located close to female nucleus in the same egg (Figure 9B). By 20 min af, the female and male pronuclei approached each other to fuse (Figure 9C), thus revealing one or two decondensed pronuclei at this time (Table 6). Metaphase of the first cleavage was detected in 30 min af (Figure 9D, Table 6). Soon thereafter, anaphase of the first cleavage was detected within 30 and 40 min af (Figure 9E, Table 6). By 50 min af, pro-metaphase of the second cleavage was observed (Figure 9F, Table 6), and in 60 min af, the embryos reached anaphase of the second cleavage (Table 6).

**Table 6:** Cytological stages of H-E stained nuclear behavior in the control and cold-shocked eggs of the loach at specific time intervals after fertilization.

Stages		Time elapsed after fertilization (min)												
		
		Control						Cold-shock						
		
		10	20	30	40	50	60	70	80	90	100	110	120	130
Second polar body release	Female nucleus (+)	13	0	0	0	0	0	2	0	0	0	0	0	0
	Female nucleus (-)	0	0	0	0	0	0	4	0	0	0	0	0	0
	Irregular division	0	0	0	0	0	0	3	0	0	0	0	0	0
	Suppression	0	0	0	0	0	0	4	0	0	0	0	0	0
2 decondensed pronuclei		0	10	0	0	0	0	0	0	3	0	0	0	0
1 decondensed pronucleus		0	6	0	0	0	0	0	3	10	0	5	0	0
First cleavage	Metaphase	0	0	14	1	0	0	0	0	0	1	0	0	0
	Anaphase	0	0	0	16	0	0	0	0	0	4	0	0	0
	Telophase	0	0	0	2	0	0	0	0	0	0	5	3	0
Second cleavage	Metaphase	0	0	0	0	22	0	0	0	0	0	0	0	1
	Anaphase	0	0	0	0	0	15	0	0	0	0	0	0	1
Anuclear or Nuclear-	1-cell	0	0	0	0	0	2	3	5	8	8	4	0	1^1^
Anuclear mosaic	2-cell	0	0	0	0	0	0	0	0	0	0	0	8	18
	3-cell	0	0	0	0	0	0	0	0	0	0	0	0	2
Others		0	0	0	0	0	0	0	0	0	3^2^	0	7^3^	2^4^
Total number of eggs observed		13	16	14	19	22	17	16	8	21	16	14	18	25

^1 ^1-cell with anuclear nucleus with four asters.

^2 ^1-cell embryo with a clumped nucleus.

^3 ^Second cleavage metaphase with a clumped nucleus (2 eggs), first cleavage metaphase with a clumped nucleus and three asters (1egg), first cleavage metaphase with a clumped nucleus and four asters (3 eggs), 1-cell with two decondensed pronuclei (1 egg).

^4 ^Second cleavage metaphase with excentric nuclear position (2 eggs).

**Figure 10:**
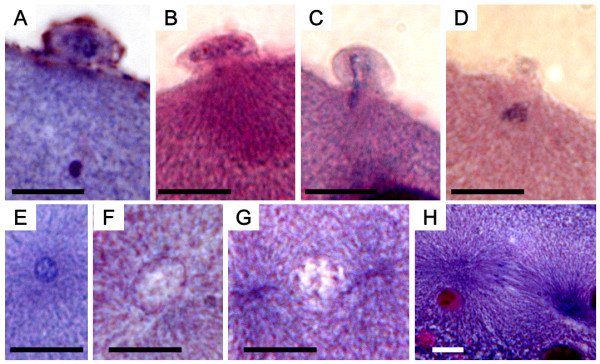
**Histological sections of cold-shocked eggs after fertilization**. A (70 min after fertilization: af): second polar body released and egg nucleus present just underneath the polar body; B (70 min af) second polar body present, but no egg nucleus; C (70 min af): abnormal extrusion of second polar body (note abnormal metaphase equator or nuclear substances ranging from egg surface to polar body); D (70 min af): extrusion of second polar body suppressed; E (70 min af): condensed sperm nucleus present; F (80 min af): decondensed male pronucleus appears; G (90 min af): two asters seen; H (100 min af): anaphase of first cleavage. Scale bars denote 10 μm.

In cold-shocked embryos, the second polar body was released within 70 min af, about 60 min later than the control (Table 6). Polar body extrusion could be categorized into four types based on histological observations: (1) the polar body was released and female (egg) nucleus was located just underneath the polar body (Figure 10A); (2) the polar body was released but no female nucleus was observed in the egg (Figure 10B); (3) the irregular metaphase equator was perpendicular to the surface of egg and nuclear material distributed from the egg surface to the polar body (Figure 10C); (4) the polar body release was suppressed and the nucleus (nuclei) remained in the egg (Figure 10D). At the same time (70 min af), a condensed nucleus, probably male nucleus, was also seen (Figure 10E), and in 10 more minutes (80 min af), a decondensed pronucleus emerged (Figure 10F). Within 90 min af, most embryos exhibited one decondensed pronucleus and the centriole replicated to form two asters or poles (Figure 10G), although two decondensed pronuclei were also detected in some eggs. Subsequently, in another 10 min (100 min af), the first cleavage occurred (Figure 10H), and by 110 and 120 min af, the first cleavage was completed resulting in two daughter nuclei (Table 6). The second cleavage followed (130 min af) (Table 6).

**Figure 11:**
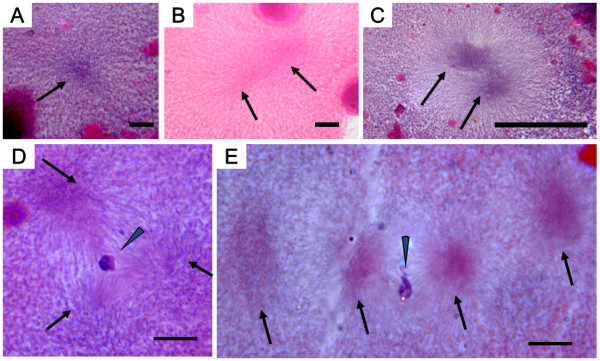
**Histological sections showing abnormalities in cold-shocked eggs after fertilization (af)**. A (80 min af) anuclear cell with one aster (arrow); B (90 min af) anuclear cell with two asters (arrows); C (100 min af) anuclear cell with two asters (arrows); D (120 min af) cell with a clumped nucleus (arrowhead) and a tripolar spindle with three asters (arrows); E (120 min af) cell with a clumped nucleus (arrowhead) and four asters (arrows). Scales denote 10 μm.

Besides the quasi-normal developing embryos mentioned above, various abnormal embryos were also observed in the cold-shocked group. The most frequent abnormality was seen in the form of anucler embryos (Table 6 Figure 11A-C), while other, less frequent, abnormalities were: a tripolar spindle configuration with a clumped nucleus and three asters (Figure 11D), spindles with a clumped nucleus and four asters (Figure 11E) and various other unusual embryos (Table 6).

## Discussion

In most fish species except for salmonids, cold- or heat-shock treatment of eggs for periods ranging from 2 to 10 min after fertilization often results in duplication of maternally derived chromosomes by inhibiting the release of the second polar body. Thus, gynogenetic diploid and wild-type triploid progeny occur in gynogenetically activated and normally fertilized eggs, respectively [2,3]. In the present study, both triploid and diploid wild-type progeny were also seen in the cold-shocked groups after normal fertilization. Triploid wild-type progeny are presumably the result of the inhibiting the release of the second polar body by cold-shock, because some triploid progeny included two maternally derived microsatellite alleles. On the other hand, wild-type diploid progeny are considered escapees from the cold-shock treatment. Gynogenetically haploid, unusual aneuploids (hypo- and hyper-diploid) and mosaics (diploid-triploid, diploid-pentaploid, diploid-aneuploid, etc.) frequently appeared among abnormal wild-type progeny. These were presumed to be "side-effects" of the cold-shock treatment on cell-division and subsequent development of the eggs.

As shown by microscopic observation of DAPI-stained nucleus and histological sections, the second polar body release, formation of female and male pronucleus, fusion of the two pronuclei, first cleavage and second cleavage occurred in the loach 10, 20, 30, 40 and 60 min after fertilization, respectively. Interestingly, although our observation of the cytological processes after fertilization in the loach were essentially same in the control as previously reported [17], this was not the case for the cold-shocked eggs, which were quite different in our study. During the cold-shock treatment, one, two or three condensed nuclei were observed. After the cold-shock treatment, histological investigation showed four types of second polar body extrusion.

Type 1 (presence of egg nucleus) where the egg nucleus transforms to female pronucleus and then fuses with male pronucleus to form the zygotic nucleus, resulting in normal diploid progeny. The histological image of this type is equivalent to DAPI-stained three condensed nuclei observed under a stereoscopic fluorescence microscope.

In type 2 (absence of egg nucleus), the egg nucleus is released from the egg together with second polar body nucleus and only the sperm nucleus remains in the egg. In other words, the polar body nucleus and the egg nucleus are both likely enclosed with the polar body and then released together. The histological image of this type is equivalent to DAPI-stained two condensed nuclei.

Type 3 (abnormal nucleus) is a transition stage that disturbs the polar body release to cause complete or partial elimination of the egg nucleus. Such a process gives rise to the type 2 category of polar body extrusion or development of aneuploid embryos.

Type 4 is the successful result of inhibition of the second polar body release and thus the polar body nucleus and egg nucleus are both enclosed in the egg, resulting in triploid progeny after fusion with the sperm nucleus. In the controls, two decondensed nuclei, i.e. female and male pronucleus, were detected, which then fused to become one decondensed pronucleus, while in cold-shocked group, eggs with two pronuclei and those with one pronucleus were both observed at essentially the same time.

The conditions of the control clearly induce normal pronuclear fusion to form diploid or triploid progeny, while the cold-shock typically results in the existence of only the male pronucleus in the eggs, giving rise to androgenetic haploid progeny. However, the cold-shock treatment also appears to have serious side effects, giving rise to clumped nucleus, unusual polypolar spindle, and anuclear embryos. Such zygotes are supposed to develop into abnormal embryos and then die. Similar side effects have also been reported in the case of salmonid embryos that were manipulated to duplicate the chromosome set with heat shock [32,33] and hydrostatic pressure shock [6,34-36].

## Conclusions

**Figure 12:**
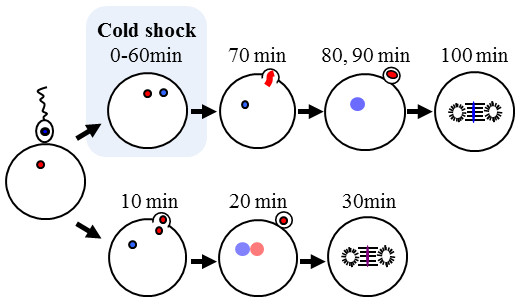
**Presumptive cytological mechanisms for androgenesis induced by cold-shock treatment for just-fertilized eggs of the loach**.

Cold-shock treatment (at 0 and 3°C) of loach eggs for 60 min just after fertilization successfully induces androgenetic haploid development at relatively high frequencies among the survivors. Successful induction of haploid androgenesis was verified by external morphology (haploid syndrome), flow cytometry (haploid DNA content), color phenotype (paternally derived recessive albino or orange phenotype) and exclusive occurrence of paternally derived alleles in microsatellite DNA genotypes of the resultant progeny. The cytological mechanisms for androgenesis induced by cold-shock are explained as follows: the egg nucleus is presumably extruded together with the second polar body by the cold-shock treatment and thereby only sperm nucleus remains in the egg. Sperm nucleus transforms to male pronucleus and then initiates androgenetic development (Figure 12).

Successful induction of androgenesis by the cold-shock may open new possibilities of chromosome manipulation. The next step in this application is the production of viable diploid androgenotes (very few survived in this study). However, the mechanism by which diploid androgenotes are generated is unknown. To obtain diploid androgenotes, chromosome duplication by inducing endomitosis at an early cleavage stage [2,3,6-9,36] and the use of diploid sperm [10-15,35-41] are required to examine androgenesis after cold-shock. Another extension of this work is the application of the cold-shock androgenesis technique to other fish species.

## Methods

### Ethics

This study was performed in accordance with the Guide for the Care and Use of Laboratory Animals in Hokkaido University.

### Fishes

Wild-type diploid loach *Misgurnus anguillicaudatus *were collected in Iwamizawa city, Hokkaido, Japan and stocked in the Aquarium center, Faculty of Fisheries Sciences, Hokkaido University, Hakodate city. Albino and orange loach strains (recessive traits) were also reared in the Aquarium center. For each cross, a single pair of loach (one wild-type female and one albino or orange male) was used.

### Cold-shock treatment

Ovulation and spermiation were induced by intraperitoneal injection of human chorionic gonadotropin (20 IU/g body weight, Aska Pharmaceutical Co. Ltd., Tokyo, Japan) as described in [42]. Eggs were collected on a polyvinylidene chloride film (SaranWrap: Asahi Kasei Co. Ltd., Tokyo, Japan) stretched over the bottom of a 90-mm diameter plastic dish. Sperm was collected into hematocrit tubes and diluted 1: 20 with Kurokura solution [43]. Eggs were mixed with diluted sperm and activated in ambient freshwater. In the first experiment to optimize cold-shock temperature among 0, 3, 6 and 9°C, five crosses (A to E) were made. Average egg number was 307.8 for control and 831.8 for cold-shock. In the second experiment to confirm effectiveness of 3°C cold-shock, six crosses (F to K) using different brood stock were made. Average egg number was 307.2 for control and 408.2 for cold-shock.

The plastic dish holding a batch of just-fertilized eggs on the film was transferred to a Styrofoam chamber containing cold freshwater with temperature adjusted to 0, 3, 6 or 9°C. After a 60 min treatment, the plastic dish was placed in another chamber containing 20°C freshwater for 210 min after fertilization. Subsequently, the eggs were incubated in the plastic dish at 20°C. A plastic dish with a batch of eggs that was transferred directly to the chamber with 20°C freshwater and placed there for 210 min after fertilization was used as control group.

### Rates of fertilization and hatching, and external appearance

Fertilization rate was calculated within 4 h after fertilization as the proportion of cleaved eggs relative to the initial number of eggs used. Hatching rate was calculated as the proportion of hatched larvae relative to the initial number of eggs used. The rates of normal and abnormal larvae were calculated relative to the total number of hatched larvae. Color phenotypes (wild-type, albino and orange) were detected based on the expression of melanophores according to the procedure given in [4].

### Ploidy determination and microsatellite genotyping

The DNA contents of hatched larvae were measured by flow cytometry based on the guidelines in [4]. Ploidy was determined by comparing the relative DNA content against a standard DNA content of the diploid loach. Genotyping was done for females, males and the progeny from control and cold-shocked groups at *Mac60, 63, 402*, and/or *519 *loci according to the procedures outlined in [31].

### Cytological and histological examination

Control and cold-shocked eggs for cytological observation were fixed with 4% paraformaldehyde dissolved in Phosphate Buffered Saline (PBS: NaCl 8 g, Na_2_HPO_4_-12H_2_O 2.9 g, KCl 0.2 g, KH_2_PO_4 _0.2 g/1000 ml double distilled water DDW) for 24 h and then transferred to PBS, then stored in a refrigerator (4°C). The chorion was mechanically removed and the blastodisc stained for 30 min in the dark with a mixture of 10 μl of DAPI (4'6-diamidino-2-phenylindole 1 mg/100 ml (DDW)), 985 μl of buffer A (Tris(hydroxyl-methyl)amino methane 0.124 g, EDTA-2Na 0.3722 g, Nacl 0.5844 g/100 ml DDW-NaCl-EDTA buffer (pH7.4)) and 5 μl of buffer B (2-mercaptoethylamine hydrochloride 0.1136 g/100 ml DDW). The samples were then rinsed twice with PBS for 10 min each, followed by one rinse for 2 min with 30% glycerol and 50% glycerol for 2 min. Stained samples were observed using fluorescence microscope (Nikon ECLIPSE E800, Tokyo, Japan).

Eggs and embryos from control and cold-shocked groups were fixed with Bouin's fixative for 3 h and the fixed samples were stored in 80% ethanol. The samples were subsequently dehydrated and embedded in paraffin blocks. Sections were cut at 8 μm thickness, stained with hematoxylin-eosin, and observed under microscope.

### Statistical analysis

Data are shown as mean ± S.D. Fertilization rates, hatching rates and phenotypic (external appearance and color trait) rates of fry classified with four types of morphological features in the first cold-shock experiment using 0, 3, 6 and 9°C cold-shock temperatures and those in the second cold-shock experiment using the selected 3°C temperature were subjected to Kruskal-Walis tests. Subsequently, statistical significance was evaluated by means of a post-hoc multiple comparison using the Scheffé test. In the latter cold-shock treatment, fertilization rates and hatching rates were subjected to Mann-Whitney's U-test. Statistical significance was set at 0.05.

## Authors' contributions

KM, KA, TF and EY designed research. KM, TF, MS and AK were in charge of fish breeding and experimental crossing. KM, MS and YZ performed molecular genotyping. TF and AK performed cytological observation. TF, KA and EY analyzed data and wrote the final drafts of the paper. All authors read and approved the final manuscript.
